# The bHLH transcription factor DEC1 promotes thyroid cancer aggressiveness by the interplay with NOTCH1

**DOI:** 10.1038/s41419-018-0933-y

**Published:** 2018-08-29

**Authors:** Cristina Gallo, Valentina Fragliasso, Benedetta Donati, Federica Torricelli, Annalisa Tameni, Simonetta Piana, Alessia Ciarrocchi

**Affiliations:** 1 Laboratory of Translational Research, Azienda Unità Sanitaria Locale - IRCCS di Reggio Emilia, Reggio Emilia, 42123 Italy; 2 Pathology Unit, Department of Oncology, Azienda Unità Sanitaria Locale - IRCCS di Reggio Emilia, Reggio Emilia, 42123 Italy

## Abstract

Aberrant re-activation of transcription factors occurs frequently in cancer. Recently, we found the basic helix-loop-helix transcription factors DEC1 and DEC2 significantly up-regulated in a model of highly aggressive thyroid cancer, raising the hypothesis that these factors might be part of the program driving progression of these tumors. Here, we investigated for the first time the function of DEC1 and DEC2 in thyroid cancer. Using both gain- and loss-of-function approaches, we showed that DEC1 more than DEC2 sustains progression of thyroid cancer by promoting cell growth and invasiveness. We demonstrated that DEC1 controls NOTCH1 expression and that the interplay with the NOTCH pathway is relevant for DEC1 function in thyroid cancer. We confirmed this observation in vivo showing that DEC1 expression is a specific feature of tumor cells, that this transcription factor is significantly over-expressed in all major thyroid cancer histotypes and that its expression correlated with NOTCH1 in these tumors. Finally, we performed RNA-sequencing to define the DEC1-associated gene expression profile in thyroid cancer cells and we discovered that DEC1 drives the expression of many cell cycle-related genes, uncovering a potential new function for this transcription factor in cancer.

## Introduction

Thyrocyte-derived cancers are the most common malignancies of the endocrine system^1^. These tumors are classified as differentiated (DTC), poorly-differentiated (PDTC), and anaplastic thyroid carcinomas (ATC)^2,3^. Aggressiveness and lethality decrease with tumor cell differentiation^4,5^. Recently we reported that the transcription regulator Id1 promotes aggressiveness of thyroid carcinomas by regulating the expression of genes involved in epithelial to mesenchymal transition (EMT), invasion, and migration^6^.

Several transcription factors (TFs) were under the control of Id1 in thyroid cancer including the basic Helix-Loop-Helix (bHLH) proteins DEC1 and DEC2^7^. DEC1 and DEC2 are members of the Hairy/E(spl)/HES subgroup within the bHLH TFs family^8–11^.

Generally, DEC1 and DEC2 are associated with transcriptional repression of target genes in collaboration with the HDACs^12^. DEC1 and DEC2 are expressed in a variety of developing and adult tissues and regulate many relevant biological functions^13,14^. DEC1 and DEC2 are induced by various stress stimuli including hypoxia, and the increased expression of DEC1 and DEC2 is associated with cell survival^15,16^. Also, DEC1 and DEC2 have been suggested to play roles in carcinogenesis, cancer development, invasion, and metastasis even if with often controversial and opposing results^17,18^.

Currently, no evidence of a role of DEC1 and DEC2 in thyroid cancer exists. However, our observation that both these factors are strongly induced in Id1 over-expressing and highly aggressive thyroid cancer cells seems to indicate that DEC1 and DEC2 may be part of a transcriptional program that promotes aggressiveness and metastatic spreading of thyroid cancer.

NOTCH1 is a member of a family of four transmembrane receptors. In the canonical activation of NOTCH1-dependent signaling, the intracellular NOTCH1 domain (NICD) is cleaved and translocates to the nucleus where in collaboration with other TFs controls gene expression^19^.

Many evidence suggested a role for NOTCH1 in carcinogenesis and tumor progression^20^. Depending on context and tumor stage, aberrant NOTCH1 signaling has been directly linked to tumor suppressor or oncogene function^21^. Also, in thyroid cancer, NOTCH1 plays a controversial and not fully defined role. Even if, activation of NOTCH pathway has been shown to restrain thyroid cancer cell proliferation^22^, NOTCH1 expression is upregulated in thyroid cancers with BRAF, RET/PTC mutations, or active MAPK signaling. In this context, activated NOTCH1 signaling promotes tumor growth^23^. Furthermore, expression of NOTCH1 has been positively correlated with papillary thyroid cancer (PTC) invasiveness and proposed as a molecular marker associated with poor prognosis^24^.

Here, we investigated the role of DEC1 and DEC2 in thyroid cancer. We also investigated the functional relationship of these TFs with NOTCH1 in the regulation of thyroid cancer biology.

## Results

### DEC1 and DEC2 are expressed in aggressive thyroid cancer models

**Fig. 1:**
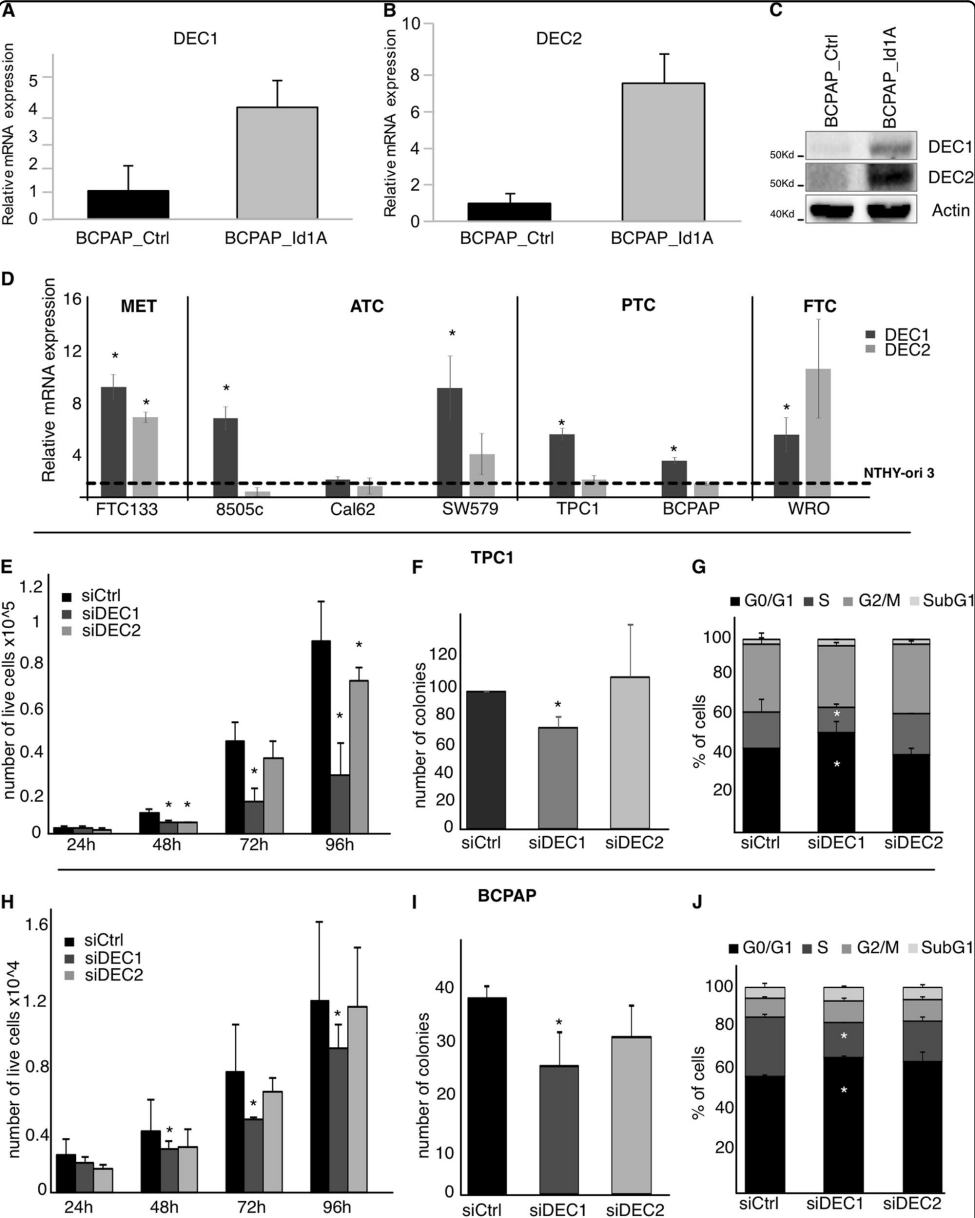
DEC1 silencing inhibits cell proliferation in thyroid cancer cell lines. **a**, **b** qRT-PCR expression of DEC1 (**a**) and DEC2 (**b**) in BCPAP-Id1A and control clones. Histograms represent the relative fold change of these factors (±SD) in BCPAP-Id1A as compared to Ctrl. **c** Western Blot analysis of DEC1 and DEC2 in BCPAP-Id1A and Ctrl clones. Actin expression was analyzed as a loading control. **d** qRT-PCR expression of DEC1 and DEC2 expression in a panel of thyroid cancer cell lines. The histograms represent the fold change ± SD of DEC1 and DEC2 expression in the indicated cell lines relative to their expression in the immortalized normal thyrocyte cell line NTHY.ori3 (broken line). **e** Cell count in TPC1 cells transfected with siRNA against DEC1, DEC2, or scramble oligos (siCtrl). The histogram indicates the number of cells ± SD for each transfection at the indicated time point. **f** Colony forming assay in TPC1 cells transfected with the indicated siRNA. The histogram represents the number of colonies for each condition. **g** Cell cycle analysis by PI staining in TPC1 cells transfected with the indicated siRNA. The histograms indicate the percentage of cells in the indicated cell cycle phases. **h** Cell count in BCPAP cells transfected with siRNA against DEC1, DEC2, or scramble oligos (siCtrl). The histograms indicate the number of cells ± SD for each transfection at the indicated time point. **i** Colony forming assay in BCPAP cells transfected with the indicated siRNA. The histogram represents the number of colonies for each condition. **j** Cell cycle analysis by PI staining in BCPAP cells transfected with the indicated siRNA. The histograms indicate the percentage of cells in the indicated cell cycle phases

Recently, we found DEC1 and DEC2 significantly upregulated in a genetically modified model of thyroid cancer that acquired feature of aggressiveness (BCPAP_Id1A)^6^. First, we confirmed these data by analyzing DEC1 and DEC2 levels by qRT-PCR and western blot in BCPAP_Id1A and parental control clones (BCPAP_Ctrl)^6^. Both DEC1 and DEC2 mRNA (Fig. 1a, b) and protein (Fig. 1c) were significantly higher in BCPAP_Id1A as compared to control. We also analyzed DEC1 and DEC2 mRNA expression in a panel of thyroid cancer cell lines. Figure 1D shows the fold change of DEC1 and DEC2 mRNA expression in FTC133 (Metastasis) 8505c, Cal62 and SW579 (ATC), TPC1 and BCPAP (PTC), and WRO (FTC) relatively to the levels of these TFs in the immortalized normal thyrocyte cell line NTHY-ori3.1. DEC1 was significantly overexpressed in all cancer cell lines analyzed with the exception of Cal62 that expressed low levels of both DEC1 and DEC2. By contrast, DEC2 expression was high only in FTC133 and WRO. Noticeably, metastatic cell line FTC133 showed the highest expression of both these TFs in line with the hypothesis that these factors are more expressed in the aggressive thyroid cancer.

### Silencing of DEC1 but not DEC2 impairs cell proliferation in thyroid cancer cells

Next, we silenced DEC1 and DEC2 by siRNAs. We performed these experiments in TPC1 and BCPAP cells that are model of PTCs since our previous data suggest a functional role of these TFs in this tumor subtype^6^. Supplementary Figure 1 shows silencing efficiency in TPC1 and BCPAP cells upon transfection of DEC1-siRNAs (Supplementary Figure 1A-C) or DEC2-siRNAs (Supplementary Figure 1D-F) as compared with scramble transfected cells (siCtrl). siDEC1 b and the siDEC2 b were used for all of the following analysis. In TPC1 DEC1, silencing resulted in a significant reduction of cell growth (Fig. 1e) and viability (Supplementary Figure 2A), while DEC2 had no evident effects. As well, silencing of DEC1 but not DEC2 impaired cell proliferation and viability of BCPAP cells (Fig. 1h and Supplementary Figure 2B). Next, colony forming assay showed that number and size of the colonies were significantly reduced upon siDEC1 transfection in TPC1 (Fig. 1f, Supplementary Figure 2C) and BCPAP (Fig. 1i) but not in siDEC2 cells (Fig. 1f, i).

To further confirm these data, we analyzed whether DEC1 silencing affects cell cycle progression in TPC1 and BCPAP (Fig. 1g, j). Noticeably, in both lines siDEC1 cells showed a 30% significant reduction of the S phase cells and a consequent accumulation of cells in G1 as compared to siCtrl cells. This observation, in line with the reduced proliferation and colony forming ability of siDEC1 cells, indicated that silencing of DEC1 impairs G1/S transition. By contrast, DEC2 silencing did not have any effect on cell cycle in both TPC1 and BCPAP cells.

**Fig. 2:**
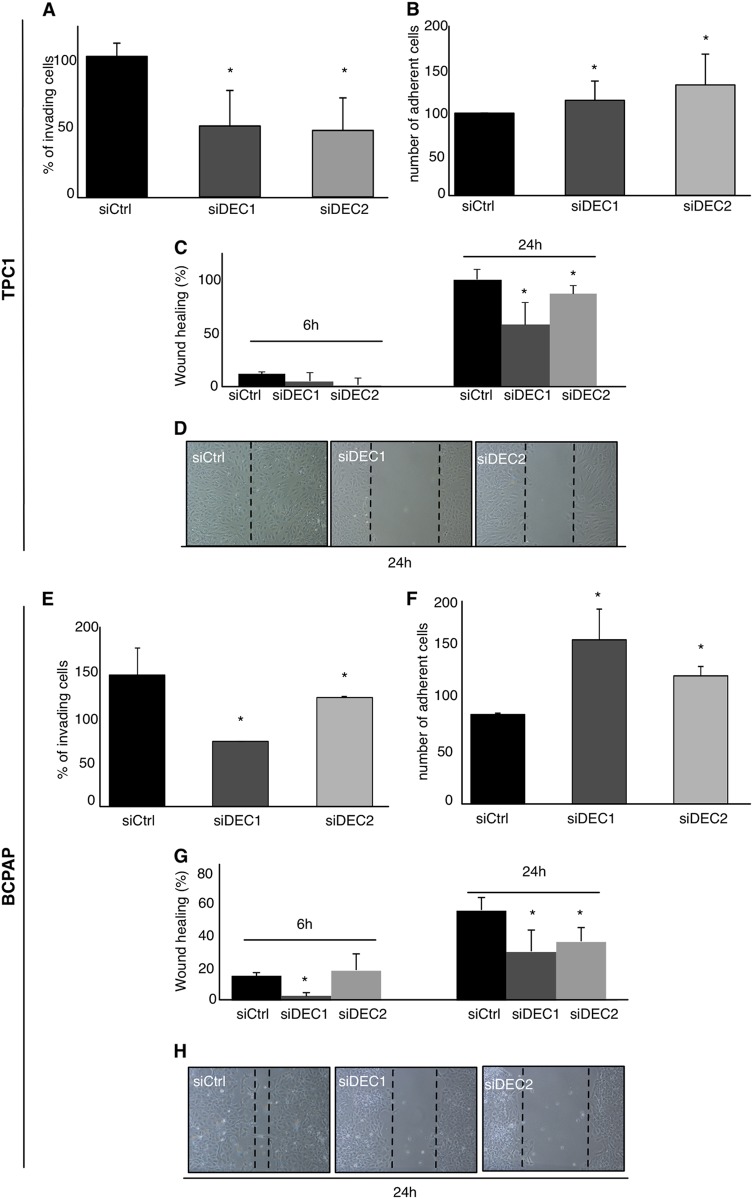
DEC1 and DEC2 silencing reduces cell migration and invasiveness of thyroid cancer cell lines. **a** Invasion assay in TPC1 cells transfected with the indicated siRNA. The histograms indicate the percentage of invading cells in siDEC1 and siDEC2 cells relative to the number of invading cells in siCtrl (100%). **b** Adhesion assay in TPC1. The histogram indicates the percentage of adherent cells in siDEC1 and siDEC2 cells relative to siCtrl (100%). **c**, **d** Wound-healing assay in TPC1 transfected with the indicated siRNA. Histogram represents the percentage of scratches healing in the indicated conditions. Magnification 200×. **e** Invasion assay in BCPAP cells transfected with the indicated siRNA. The histograms indicate the percentage of invading cells in siDEC1 and siDEC2 cells relative to the number of invading cells in siCtrl (100%). **f** Adhesion assay in BCPAP. The histogram indicates the percentage of adherent cells in siDEC1 and siDEC2 cells relative to siCtrl (100%). **g**, **h** Wound-healing assay in BCPAP transfected with the indicated siRNA. Histograms represent the percentage of scratches healing in the indicated conditions. Magnification 200×

Then, we evaluated the effects of DEC1 and DEC2 knock-down on invasion and migration. Silencing of either DEC1 or DEC2 in both TPC1 and BCPAP, resulted in a reduced ability of cells to invade (Fig. 2a, e). In agreement with this observation, silencing of DEC1 or DEC2 increased adhesion properties of both TPC1 and BCPAP (Fig. 2b, f) as compared with siCtrl cells. Finally, scratch wound-healing assay showed that silencing of DEC1 or DEC2 inhibits migration in both TPC1 and BCPAP (Fig. 2c, d, g, h). These results indicate that even if both TFs are important DEC1 plays a more relevant role than that of DEC2 in sustaining thyroid cancer progression.

### DEC1 overexpression induces thyroid cancer proliferation and promotes invasiveness

**Fig. 3:**
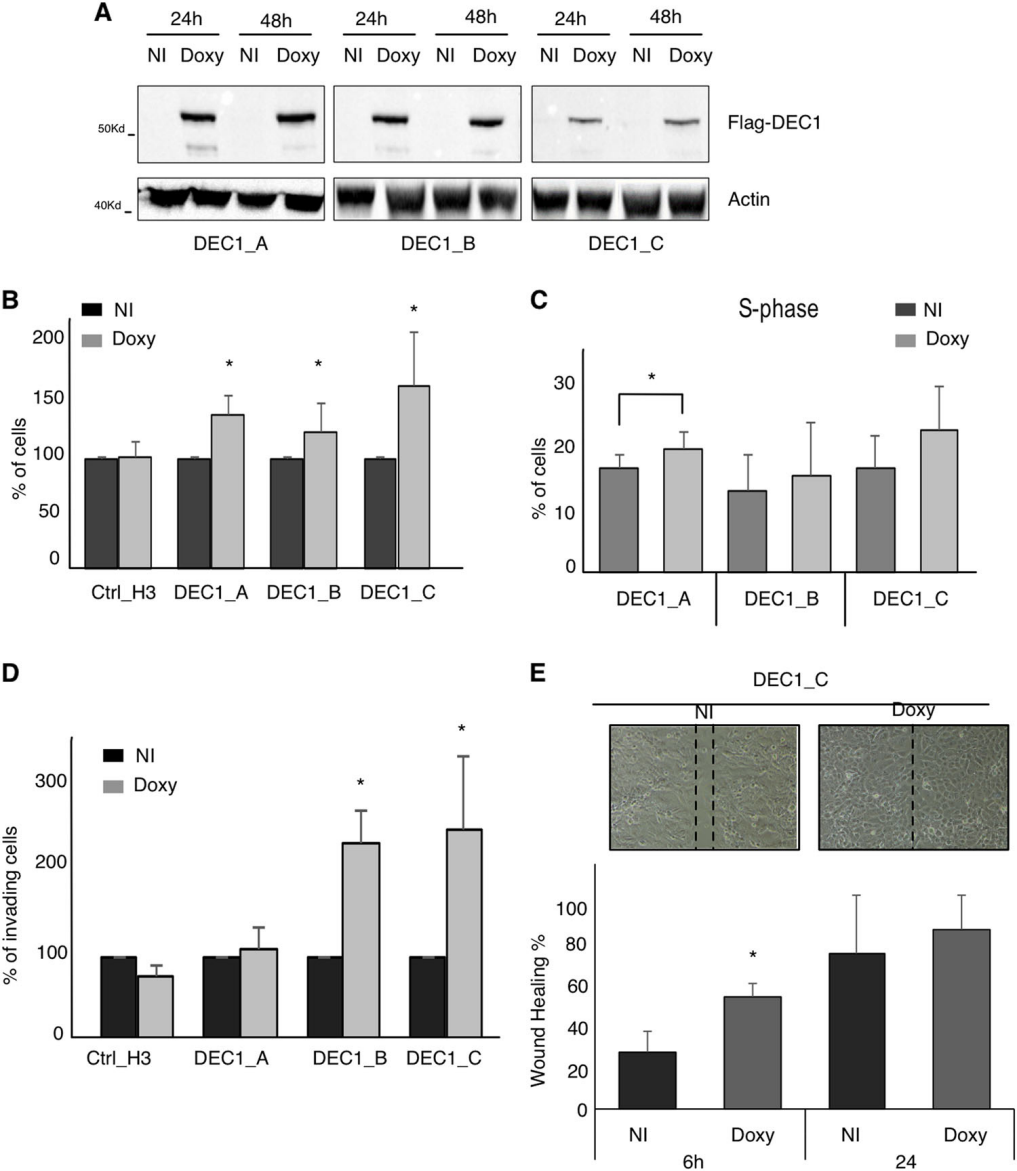
DEC1 overexpression increases proliferation and aggressiveness of thyroid cancer cells. **a** Western blot analysis of Flag-DEC1 expression in three TPC1-derived DEC1 overexpressing clones. Induction of Flag-DEC1 expression with or without doxycycline induction is shown at the indicated time point. Western blot for beta-actin is used as loading control. **b** Cell count in the three DEC1 overexpressing clones and of the Ctrl-H3 control clones with or without doxycycline induction. Histograms indicate the cell percentage fold change in doxycycline-stimulated cells as compared to non-induced (NI) cells. **c** Cell cycle analysis by PI staining in three DEC1-overexpressing clones with or without doxycycline stimulation. Histograms represent the percentage of cells in S phase in the indicated condition. **d** Invasion assay in DEC1 overexpressing clones in the presence or absence of doxycycline stimulation. Histograms represent the percentage of invading cells in doxycycline-induced cells as compared to non-treated cells (NI). **e** Scratch wound-healing assay in DEC1_C clone with or without doxycycline. Histograms represent the percentage of healing in two conditions after 6 and 24 h

To confirm this hypothesis, we overexpress DEC1 in thyroid cancer cells. We cloned DEC1, in fusion with a Flag epitope at the N-terminal, in the pSG213 expression vector, under control of a doxycycline-inducible promoter. This construct was transfected into BCPAP cells and stable clones were derived by antibiotic selection. Three DEC1 clones were chosen for further analysis: BCPAP DEC1_A, DEC1_B, and DEC1_C. Figure 3a shows the levels of Flag-DEC1 expression upon doxycycline induction.

Cell proliferation analysis showed that doxycycline stimulation significantly increased cell proliferation in all DEC1 clones, while had no effect on proliferation of control cells (Ctrl-H3) (Fig. 3b). In accordance, cell cycle analysis showed that DEC1 overexpressing clones displayed a trend toward a higher S-phase entry, which was particularly evident in the DEC1A clone (Fig. 3c). Next, we assessed the effect of DEC1 overexpression on invasion and migration of thyroid cancer cells. All three DEC1 overexpressing clones, showed increased invasiveness upon doxycycline induction, while this effect was not visible in Crtl-H3 clone (Fig. 3d). By contrast, overexpression of DEC1 seemed had a marginal effect on cell migration, as determined by scratch wound-healing assays (Fig. 3e). Overall, these data confirmed the results obtained by DEC1 silencing and support the hypothesis that DEC1 plays a significant role in controlling aggressive features in thyroid cancer.

### DEC1 expression in vivo is induced in tumor cells and characteristic of both well differentiated and undifferentiated thyroid cancers

Next, we analyzed DEC1 protein expression in a retrospective cohort of 54 thyroid cancers representative of the four major histotypes: ATCs (*n* = 9), PDTC (*n* = 6), PTCs (*n* = 28), and follicular thyroid cancer (FTCs; *n* = 11) (Table 1). We also included follicular adenomas in the analysis (FAs; *n* = 5).

**Table 1:** Clinico-pathological features

Clinico-pathological features	Total
	*N* = 54
Age	
<45	16
≥45	38
Gender	
Female	37
Male	17
Histological diagnosis	
PTC	28
FTC	11
PDTC	6
ATC	9
Stage TNM	
I	34
II	6
III	
IV	13
NA	1
Tumor size (cm)	
≤5	44
>5	9
NA	1
Metastasis	
Yes	17
No	37
Metastasis site	
Single	11
Multiple	6
NA	37
**DEC1 IHC**	
** ≤30**	**14**
** 30–50**	**4**
** >50**	**34**
** NA**	**2**

**Fig. 4:**
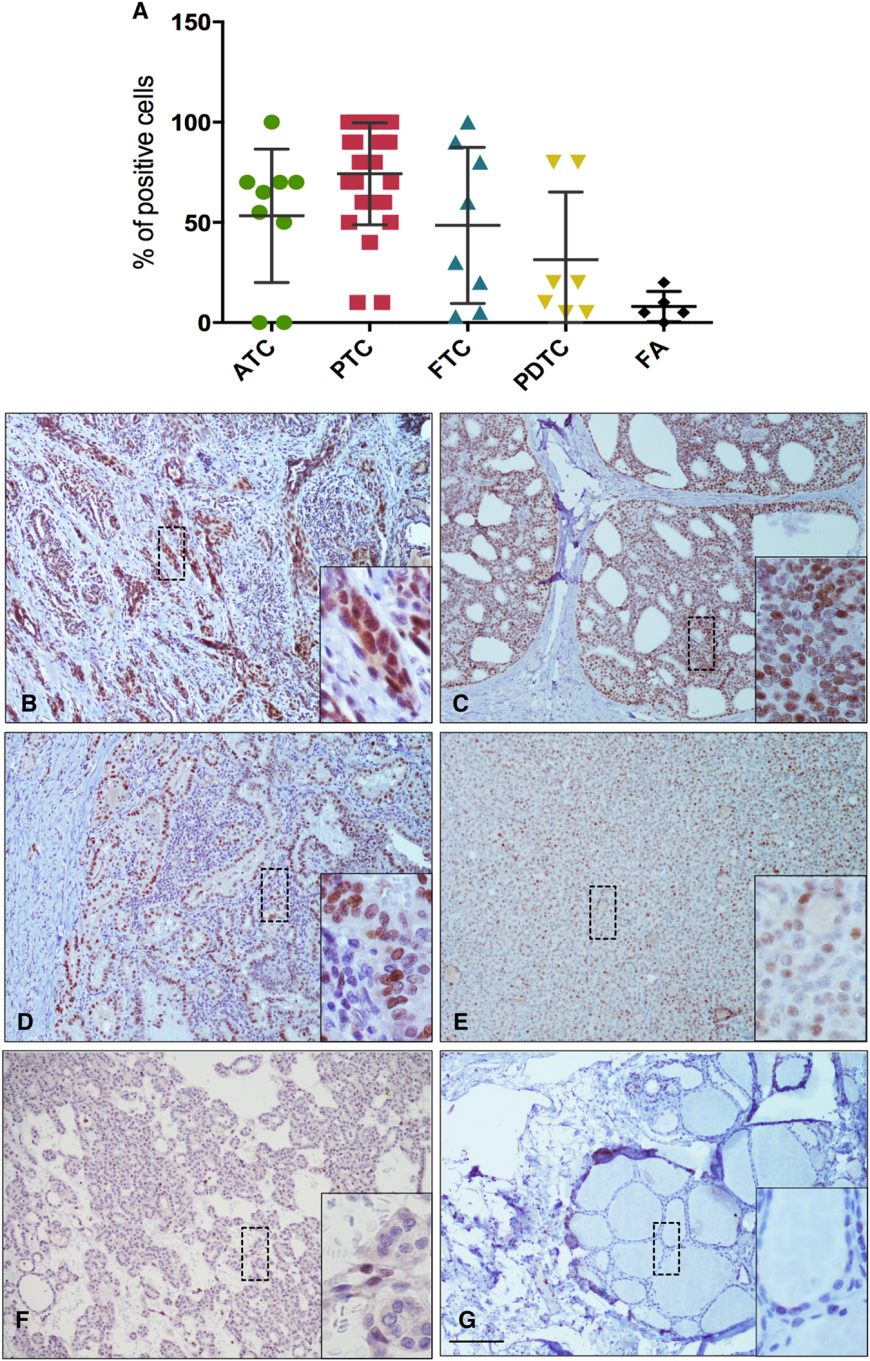
DEC1 expression in thyroid cancer patients. **a** Distribution of DEC1 positivity in the indicated thyroid cancer histotypes. The averaged percentage ± SD of positive cells in each cohort is shown. **b**–**f** Representative images of DEC1 staining in ATC (**b**), PDTC (**c**), PTC (**d**), FTC (**e**), FA (**f**), and normal thyroid (**g**)

The large majority of malignant lesions (92.5%, 50/54) displayed a strong expression of DEC1 (Fig. 4a–e). DEC1 expression was specific of tumor cells, since normal adjacent thyroid tissue scored negative for DEC1 expression in all samples (Fig. 4g). In addition, 4 out of 5 FA tested were also positive for DEC1 but the percentage of positive cells was significantly lower than that observed in malignant lesions (Fig. 4f). Supplementary Table 1 reports a correlation of DEC1 expression with major clinico-pathological features of analyzed lesions.

In all samples, DEC1 showed a nuclear localization and a homogenous expression (Fig. 4b–e). In some cases, we observed an increased concentration of DEC1 positive cells around pre-necrotic area suggesting that nutrient and oxygen deprivation may trigger DEC1 expression (Supplementary Figure 3). This is in agreement with the reported observations that DEC1 expression is induced under stress conditions and in particular under hypoxia. DEC1 expression was consistently overexpressed in all histotypes without significant differences (Fig. 4a). Noticeably, DEC1 was strongly expressed also in ATCs which represent the most aggressive and ominous form of thyroid cancer. Overall these observations confirm in vivo the relevance of DEC1 in thyroid development and progression.

### The NOTCH pathway cooperates with DEC1 to support thyroid cancer progression

Previous studies indicated a functional cooperation of NOTCH1 with bHLH Class E TFs^25–27^, suggesting a possible interplay between DEC1 and NOTCH pathway. First, we evaluated the mRNA expression profile of four members of the NOTCH family (NOTCH1–4) in thyroid cancer cell lines (Supplementary Figure 4A). We observed that NOTCH1 as DEC1 was overexpressed in cancer cell line as compared to normal thyrocytes. With the exception of FTC133, NOTCH1 expression was coherent with DEC1 expression levels. NOTCH2 was slightly upregulated in three of the analyzed cancer cell lines as compared to control, while NOTCH3 and NOTCH4 were variable and low expressed in PTC cell lines TPC1 and BCPAP.

**Fig. 5:**
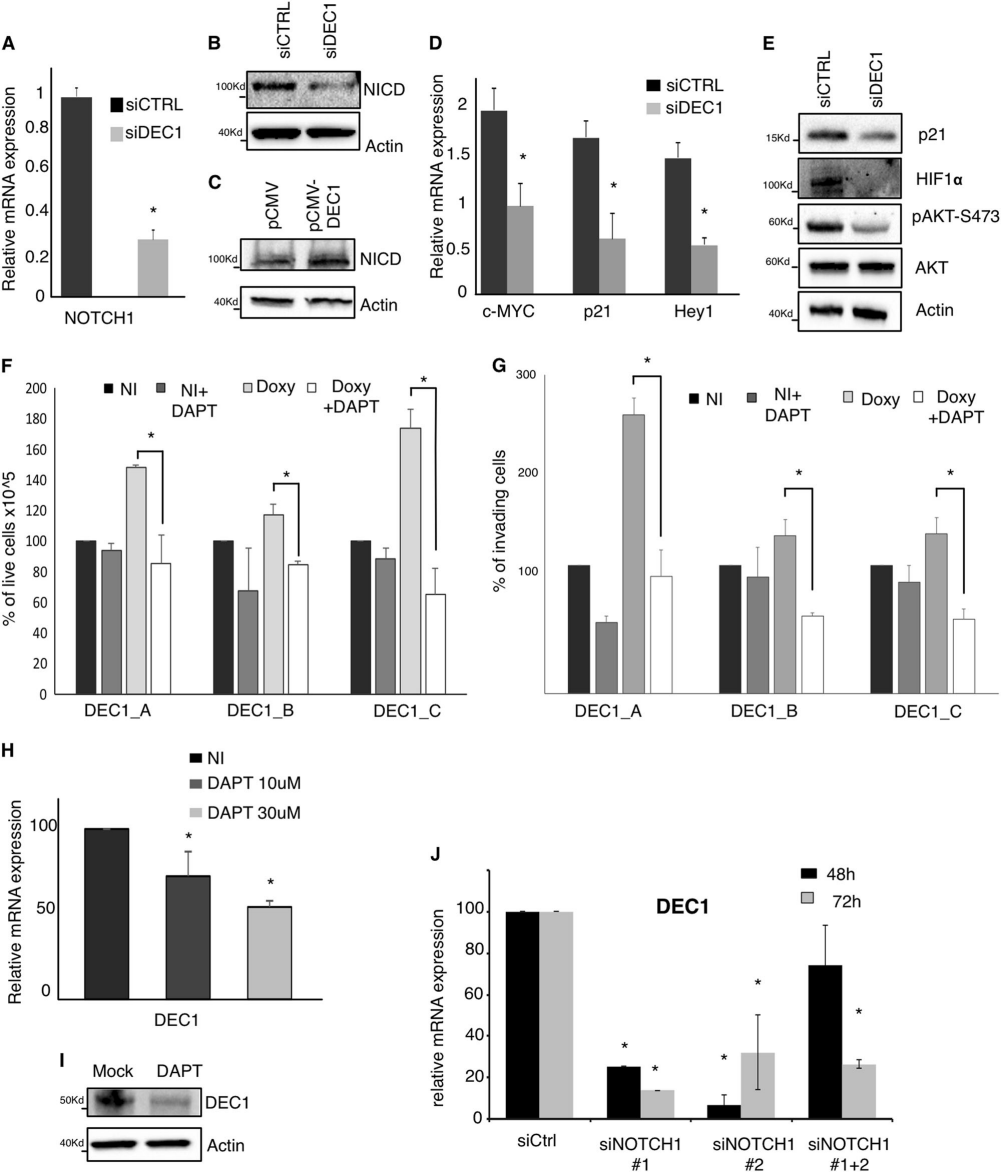
DEC1 functional interplay with the NOTCH1 pathway. **a**, **b** qRT-PCR analysis (**a**) and Western blot analysis (**b**) of Notch1 expression in DEC1 silenced or control TPC1 cells. **c** Western blot analysis of NOTCH1 in TPC1 cells transfected in transient with a DEC1 overexpressing plasmid. Western blot for beta-actin was used as loading control. **d**, **e** qRT-PCR and western blot analysis of NOTCH target-genes in DEC1 silenced or control cells. **f** Analysis of cell proliferation in DEC1 overexpressing clones upon DAPT treatment. The histograms represent the percentage of cells in the indicated conditions relatively to NI samples 72 h after DAPT treatment. **g** Analysis of cell invasion in DEC1 overexpressing clones upon DAPT treatment. The histogram represents the percentage of invading cells in the indicated conditions relatively to NI samples **h**, **i** qRT-PCR (**h**) and western blot analysis (**i**) of DEC1 expression in TPC1 cells treated or not with DAPT. **j** qRT-PCR of DEC1 expression in TPC1 cells transfected with NOTCH1 specific siRNA or with scramble oligos

Next, to investigate a possible cooperation between DEC1 and NOTCH1, we analyzed the effect of DEC1 silencing on the expression and activation of NOTCH pathway in TPC1 cells. We showed that both mRNA and protein expression of NOTCH1 was significantly downregulated in DEC1 KD cells (Fig. 5a, b). Vice versa, transient overexpression of DEC1 resulted in increased protein levels of the activated form of NOTCH1 (NICD) (Fig. 5c). In addition, we also observed that the mRNA expression of NOTCH1 ligands (Delta1 and Jagged1) was also repressed upon DEC1 silencing (Supplementary Figure 4B).

To confirm the positive role of DEC1 on NOTCH pathway, we checked the effect of DEC1 silencing on some established NOTCH targets^19,28,29^. qRT-PCR (Fig. 5d) and western blot analysis (Fig. 5e) demonstrated that c-MYC, p21, Hey1, and HIF1α were inhibited upon DEC1 silencing. Furthermore, reduced DEC1 expression resulted in an evident decrease in the Ser473 phosphorylation of AKT, which is induced upon NOTCH activation^30^ (Fig. 5e). Next, we investigated whether activation of the NOTCH pathway is functionally relevant for the DEC1-mediated thyroid cancer aggressiveness. To this purpose, we treated TPC1 cells with DAPT a chemical inhibitor of NOTCH cleavage and activation. First, we confirmed the inhibitory activity of DAPT on NOTCH pathway in our system by assessing protein expression of NICD, p21, and HIF1α (Supplementary Figure 4C). We also observed that DAPT treatment slightly inhibits cell proliferation and viability of both TPC1 and BCPAP in a dose-dependent manner, as we had already observed for DEC1 inhibition (Supplementary Figure 5A-D). To confirm that these effects were mediated by inhibition of NOTCH1 and not by unspecific DAPT effects we performed silencing of NOTCH1 in TPC1 cells using two independent siRNA. Both siRNAs used and their combination resulted in a significant inhibition of NOTCH1 levels (Supplementary Figure 5E-F). We showed that silencing of NOTCH1 resulted in a significant inhibition of TPC1 cell growth, similarly to the effect observed with DAPT (Supplementary Figure 5G).

Next, we treated DEC1-A, DEC1-B, and DEC1-C clones with DAPT and we evaluated changes in cell proliferation and invasiveness (Supplementary Figure 5H). Noticeably, NOTCH1 inhibition completely abolished the gain in cell proliferation induced by DEC1 overexpression in all clones (Fig. 5f). As well DAPT treatment impaired the DEC1 positive effect on cell invasion (Fig. 5g)

Altogether these observations indicate that: (1) DEC1 promotes the activation of the NOTCH pathway and (2) activation of the NOTCH pathway is necessary for the tumor-promoting function of DEC1 in the thyroid. Noticeably, we also demonstrated that DAPT treatment as well as NOTCH1 silencing reduced both mRNA and protein levels of DEC1 (Fig. 5h–l). To the best of our knowledge, this is the first report describing DEC1 as target of NOTCH1. This observation implies the existence of a possible positive feedback between DEC1 and NOTCH1 that would further fuels thyroid cancer development and progression.

To further consolidate these observations, we analyzed the possible correlation between DEC1 and NOTCH1 expression in human thyroid cancer samples.

NOTCH1 protein expression was analyzed by IHC in 43 of the 54 thyroid cancer samples in which DEC1 was previously analyzed: ATCs *n* = 4, PDTC *n* = 5, PTC *n* = 27, FTC *n* = 6 (Supplementary Figure 6A-F). Five FAs were also analyzed. In accordance with the wide DEC1 expression observed in these lesions, NOTCH1 expression was positive in 93.02% (40/43) of tested malignant lesions. NOTCH1 staining was mainly localized at the membrane of cancer cells and in some cases limited to an apical localization. Furthermore, as previously reported, NOTCH1 also marked blood vessels. We did not observe expression of NOTCH1 in normal thyrocytes while a limited expression of this protein was observed in FAs where NOTCH1 positivity was prevalently limited to vessels. Correlation analysis of DEC1 and NOTCH1 expression levels in these lesions showed that these factors were significantly associated in thyroid malignant lesions (Table 2, Supplementary Figure 6G-H), and that DEC1 tended to be highly expressed than NOTCH1 in ATC. These observations confirm our in vitro analysis and further highlight the existence of a functional interplay between DEC1 and NOTCH1 in thyroid cancer.

**Table 2:** Correlation analysis of DEC1 and NOTCH1 expression in thyroid cancer samples

**NOTCH1 IHC**	DEC1 IHC		
	≤30	30–50	>50
**≤30**	**7**	**1**	**7**
**30–50**	**1**	**0**	**7**
**>50**	**1**	**3**	**15**

*p*-Value = 0.03; calculated by Fisher’s exact test

### DEC1 coordinates the expression of a cell cycle-related genes

**Fig. 6:**
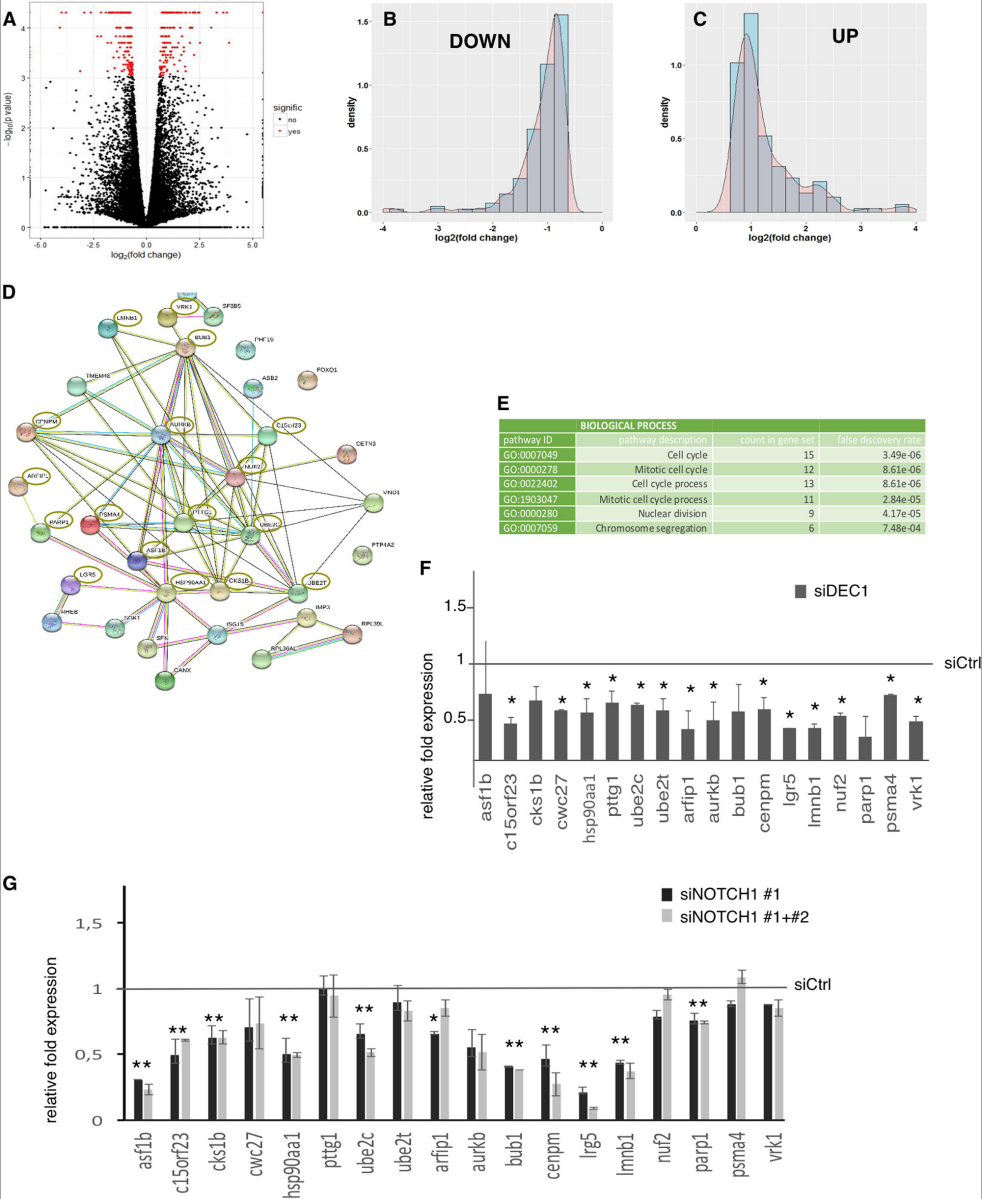
**a** Volcano Plot showing the distribution of RNA-Seq data. **b**, **c** Histograms representing the distribution of log2 fold change. **d**, **e** Protein–protein interaction network showing DEC1 target genes involved in cell cycle regulation and DNA stability. **f** qRT-PCR validation analysis of a panel of DEC1 target genes within the network shown in (**c**). Histogram represents the relative fold change ± SD of these genes in DEC1 silenced TPC1 cells as compared to control cells (set as 1, black line). **g** qRT-PCR analysis of selected DEC1 target genes in TPC1 cells transfected with NOTCH1 #1 or NOTCH1#1+2 siRNA or scramble siRNA as control. Histogram represents the relative fold change ± SD of these genes in NOTCH1 silenced TPC1 cells as compared to control cells (set as 1, black line)

To get insights into DEC1 function in thyroid cancer, we explored the gene expression profile associated with this TF by RNA-sequencing analysis on TPC1 cells transfected with siRNA against DEC1 or Ctrl siRNA. 320 genes resulted differentially expressed in siDEC1 cells compared to controls (Fig. 6a–c). A similar number of genes were repressed (*n* = 166) or up-regulated (*n* = 154) upon DEC1 silencing. DEC1 biological functions have been associated with its transcriptional repressor activity. By contrast, these data suggest that DEC1 transcriptional activation plays a major part in mediating DEC1 effect in thyroid cancer. Next, we performed an enrichment analysis to establish which pathways were majorly affected by DEC1 silencing.

Gene Ontology analysis showed that upregulated genes were enriched in pathways related to developmental process regulation, extracellular matrix, collagen regulation, and blood vessel formation (Supplementary Figure 7A, B).

By contrast, no significant enriched pathways were detected in the down-regulated genes. However, protein network analysis using String software revealed that a subgroup of 33 down-regulated DEC1 target genes were linked within a common functional network defining a DEC1-dependent gene module in thyroid cancer (Fig. 6d). Noticeably, these genes were involved directly or indirectly in the cell cycle regulation (in particular in the G1/S transition or in the M phase) and chromosomal segregation (Fig. 6e). The observation that cell cycle-related genes were repressed upon DEC1 silencing is in agreement with the reduced proliferation and cell cycle progression observed in the functional assays and unveils a previously unknown function of DEC1 in cancer. qRT-PCR analysis confirmed the validity of the RNA-SEQ (Fig. 6f).

Noticeably, both NOTCH1 and p21 resulted downregulated in this analysis (Supplementary Figure 7C). Besides, 24 (14.4%) of the DEC1 down-regulated genes and 26 (16.8%) of the DEC1 up-regulated genes were also known to be linked with NOTCH pathway. To further support the functional interplay between NOTCH1 and DEC1, we analyzed the mRNA expression of DEC1 target genes in TPC1 upon silencing of NOTCH1. Noticeably, over 60% (11 out of 18) of analyzed target genes were also coherently affected by NOTCH1 silencing, suggesting that these genes are also NOTCH1 targets and further supporting the functional interplay between DEC1 and NOTCH1 in thyroid cancer (Fig. 6g).

## Discussion

Aberrant re-activation of TFs occurs consistently in cancer where they serve to either promote the expression of cancer driver genes or counteract suppressive pathways. In this work, we characterize the role of DEC1 in thyroid cancer.

DEC1 has been reported to have multifaceted roles in cancer progression^14^. Previous studies showed that DEC1 could repress transcription in a HDAC-dependent manner^12^, causing cell growth arrest, pro-apoptotic effects, and mediating DNA damage-induced senescence^31,32^. DEC1 also plays a role in cell survival^33^. Detrimental stimuli, including hypoxia, induce a rapid and robust up-regulation of DEC1 in cancer cells and this TF has been shown to participate to TGF-β-induced cell survival in breast cancer cells, as well as to resistance to oxidative stress-mediated cell death^34^. Furthermore, DEC1 have been shown to mediate the p53-dependent cell survival vs. cell death in response to DNA damage stress through the regulation of MYC-1^35^. Here, we demonstrated that DEC1 supports thyroid cancer proliferation mainly by promoting cell cycle and a faster S-phase entry.

In line with the complex function of DEC1, Bi et al. reported that DEC1 inhibits breast cancer cell cycle progression, however through mechanisms that are independent by its transcriptional activity^36^. These authors demonstrated that DEC1 binds to and stabilizes Cyclin E, resulting in a prolonged S phase and suppression of breast cancer cells growth. By contrast, here we showed that the cell cycle promoting function of DEC1 in thyroid cancer relies on its ability of inducing the expression of a panel of genes directly or indirectly involved in G1/S transition or mitotic exit.

Again in contrast with the “canonical” function of these TFs, we showed that the role of DEC1 in promoting thyroid cancer aggressiveness relies on the ability of DEC1 to transactivate gene expression. Basically, an equal number of genes were found induced and repressed upon DEC1 silencing in our analysis and induced genes were predicted to be highly relevant in the cell proliferation regulatory function of DEC1.

Our observation that DEC1 works as a transcriptional activator is in agreement with other reports. For example, Li et al. reported that in tumors cells DEC1 protects against apoptosis induced by serum starvation by transcriptionally inducing the expression of Survivin^37^. Two Sp1-binding sites, located adjacently to the DEC1 bounded E-boxes are required for the DEC1-dependent activation of Survivin expression suggesting that the “non-canonical” cooperation of DEC1 with Sp1 is fundamental for this function and supporting the hypothesis that DEC1-mediated transcriptional activation follows non-canonical and context-specific mechanisms. Intriguingly, we found DEC2 among genes that were altered by DEC1 silencing in thyroid cancer cells. This is not the first report indicating that DEC1 negatively controls DEC2^14,38^. However, the significance of this regulation is still to be clarified. In several tumor types, DEC2 and DEC1 have been shown to play opposite functions^13,14,31,32^. In these cases, the negative regulation on DEC2 expression may be the reflection of the contrasting function of these TFs.

Here, we evaluated for the first time the expression of DEC1 in a cohort of thyroid tumors with different histotypes. We demonstrated that DEC1 expression is specific of tumor cells and not detectable in normal thyroid cells. This is consistent with the report of Li et al. who have previously reported that DEC1 is strongly associated with tumor in a comparative tissue microarray analysis among different paired tumor-normal tissues^37^. Furthermore, we observed a strong DEC1 expression in ATCs which represent the most aggressive form of thyroid cancer. This finding is consistent with the evidence that DEC1 is highly expressed, gastric cancer with poor histologic differentiation^39^.

Finally, here we showed that DEC1 controls the expression of NOTCH1 and consequently the activation of the NOTCH pathway in thyroid cancer. We also showed that this activation is relevant in mediating the pro-cancer function of DEC1 and that in turn NOTCH1 pathway induces activation of DEC1 expression in a positive feedback loop. We also showed for the first time that DEC1 silencing is associated with decreased expression of HIF1α, which is one of the most characterized targets of the NOTCH pathway^19^. This observation is of particular relevance since DEC1 is known to be induced under hypoxia in a HIF1α depending fashion^40^. Beside, HIF1α is also known to control activation of NOTCH1 pathway in a further complicated loop of regulation^41,42^. How DEC1 controls HIF1α expression needs to be clarified by further experimental evidence. However, it is possible that the effect of DEC1 on HIF1α is indirect and mediated by the activation of the NOTCH1 pathway. Still these observations indicate that DEC1 is part of an intricate network of signals that is likely activated under stressful condition (like hypoxia) that aims to support cancer cell survival. In conclusion, this work describes for the first time a role of DEC1 in thyroid cancer, demonstrating in vitro and in vivo that this TF is important and plays a positive function during thyroid cancer tumorigenesis.

## Methods

### Cell lines and cell culture medium

TPC1 and BCPAP human cell lines were obtained from Dr. Massimo Santoro, University of Naples (Naples, Italy). These cell lines were derived from PTC samples and were cultured at 37 °C in 5% CO_2_ in DMEM medium supplemented with 10% heat-inactivated fetal bovine serum (or TET-free FBS for culturing clones). Subcloning Efficiency DH5α Competent Cells and One shot Stbl3 were used for cloning and were purchased from Invitrogen. BCPAP_Id1A and controls were derived as previously described^6,43^. Briefly, these cells were obtained as stable clones from BCPAP transfected with pCDNA3.1-Id1 or pCDNA 3.1 as control. BCPAP-Id1A express high levels of Id1 and display features of high aggressiveness.

### Immunohistochemistry

Fifty-four thyroid cancer samples were selected from the Thyroid Tumor Archive of our Institution (Table 1). Immunohistochemistry was performed as previously described^43^. Briefly, samples were derived from a cohort of thyroid cancer patients belonging to different histotypes and collected at our Institution in the Pathological Anatomy archive. Immunohistochemistry was performed on 4 μm formalin-fixed, paraffin-embedded tissue sections with a 1:300 dilution of rabbit polyclonal anti-human DEC1 antibody (Bethyl Laboratories, Inc. Montgomery, TX) and NOTCH1 antibody (D1E11-Cell Signaling) for 32′ at 37°. The tissue sections were pre-treated with heat antigen retrieval in EDTA buffer for 72′. The staining was developed on platform Ventana Bench-Mark ULTRA using the OptiView DAB Detection Kit (Ventana-Roche, Tucson, AZ). Slides were counterstained with hematoxylin.

This project was approved by the Ethical Committee of Azienda Unità Sanitaria Locale (AUSL) - IRCCS of Reggio Emilia.

### RNA-Seq analysis

RNA quality was assessed by Bioanalyzer using Agilent RNA 6000 nano kit and quantified by Nanodrop. RNAseq libraries were prepared starting from 1 µg of RNA. Next-generation sequencing was performed using NextSeq 500 platform (Illumina) and a minimum of 20 million sequencing reads for each sample replicate was expected.

Bioinformatic analysis was conducted applying Cufflink RNA-Seq workflow. Differential gene expression was calculated as fold-change (siDEC1/siCtrl). Genes with a *Q*-value < 0.05 were considered significantly deregulated. *Q*-value was obtained adjusting *p*-value by an optimized FDR approach (FDR cutoff = 0.05). Data are available at ArrayExpress accession number E-MTAB-6394.

### Statistical analysis

Data are expressed as means ± SD. The statistical significance between multiple data sets was determined by one-way ANOVA, whereas *T*-test was used for comparing two group of values, using Graph-Pad Prism software. For DEC1 and NOTCH1 expression correlation in patients, the percentages of positive cells were dichotomized into three groups (<30%, 30–50%, >50%). A contingency table of the counts for each combination of factors has been built and *p*-value has been calculated by Fisher’s exact test for count data. Comparisons have been considered significant when *p*-value was lower than 0.05. All the statistical analysis were performed using R statistical software package version 3.4.3 (R Foundation for Statistical Computing, Vienna, Austria).

## Electronic supplementary material

Supplementary Information
