# Supplementary material for: The bHLH transcription factor DEC1 promotes thyroid cancer aggressiveness by the interplay with NOTCH1
Source: Cell Death Dis. 2018 Aug 29;9(9):871. doi: 10.1038/s41419-018-0933-y (PMC6115386; doi:10.1038/s41419-018-0933-y)
Supplement: Supplementary file 1 — Supplementary Information [file 41419_2018_933_MOESM1_ESM.pdf]

Supplementary Figure 1

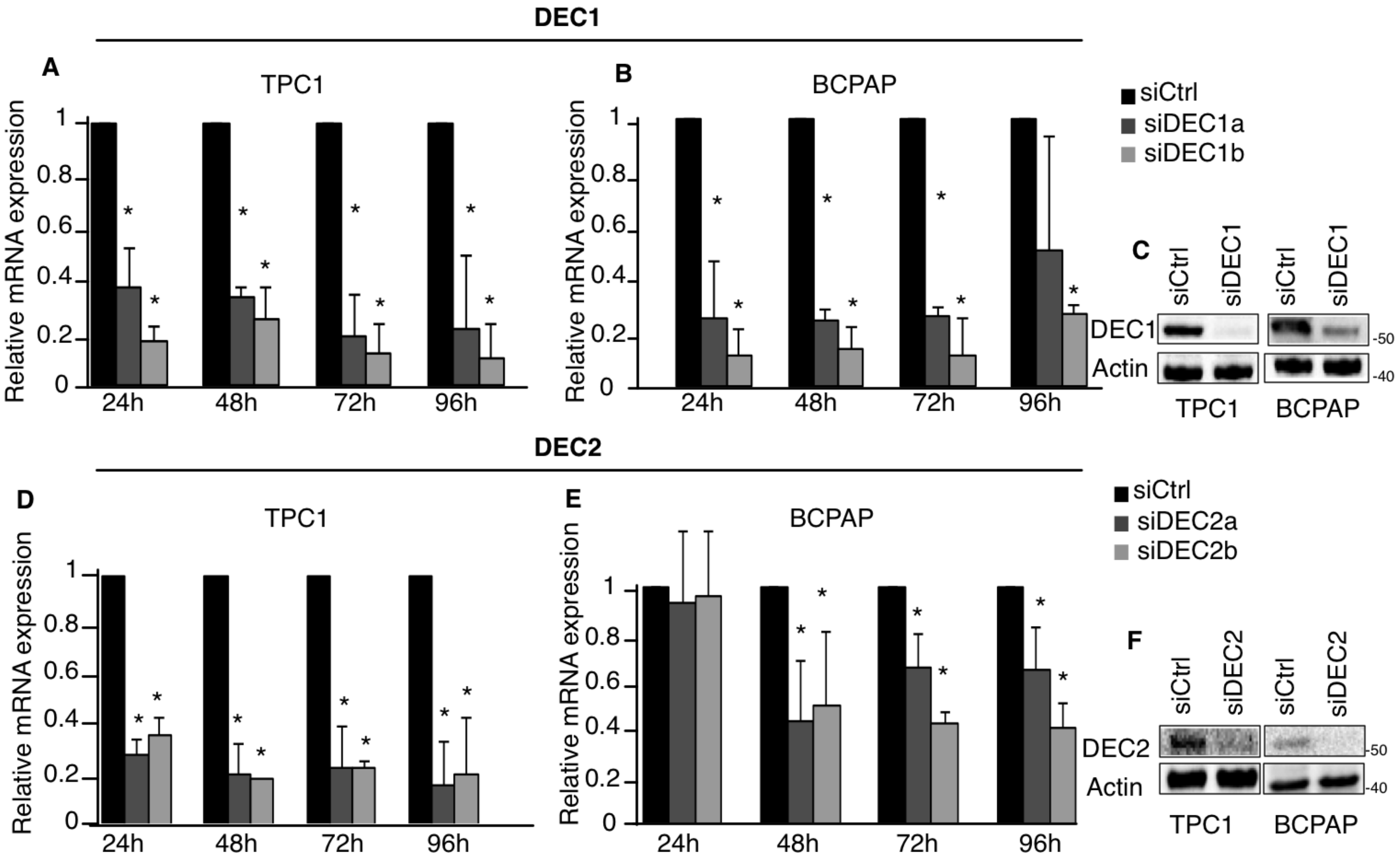

Supplementary Figure 2

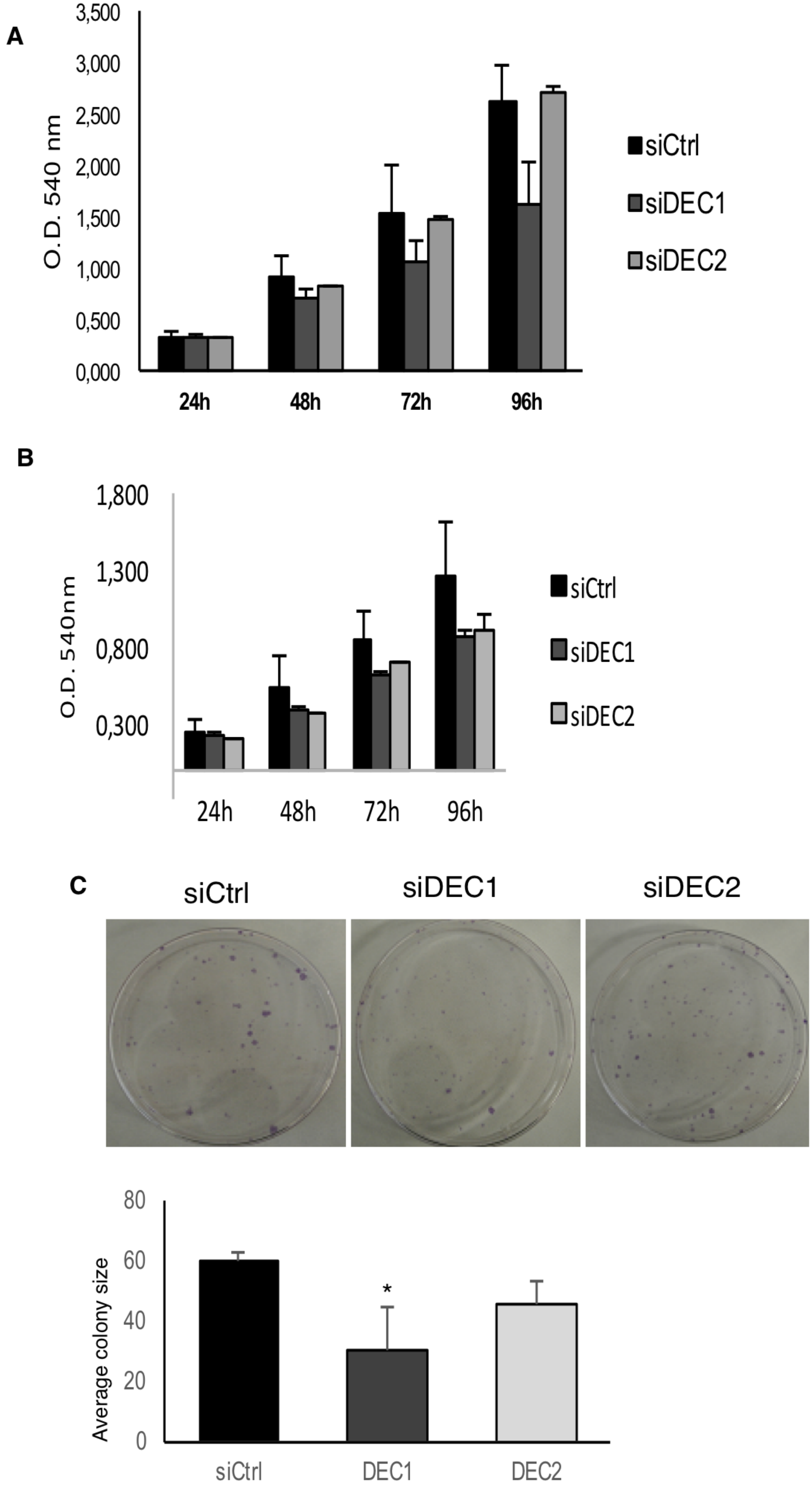

Supplementary Figure 3

A

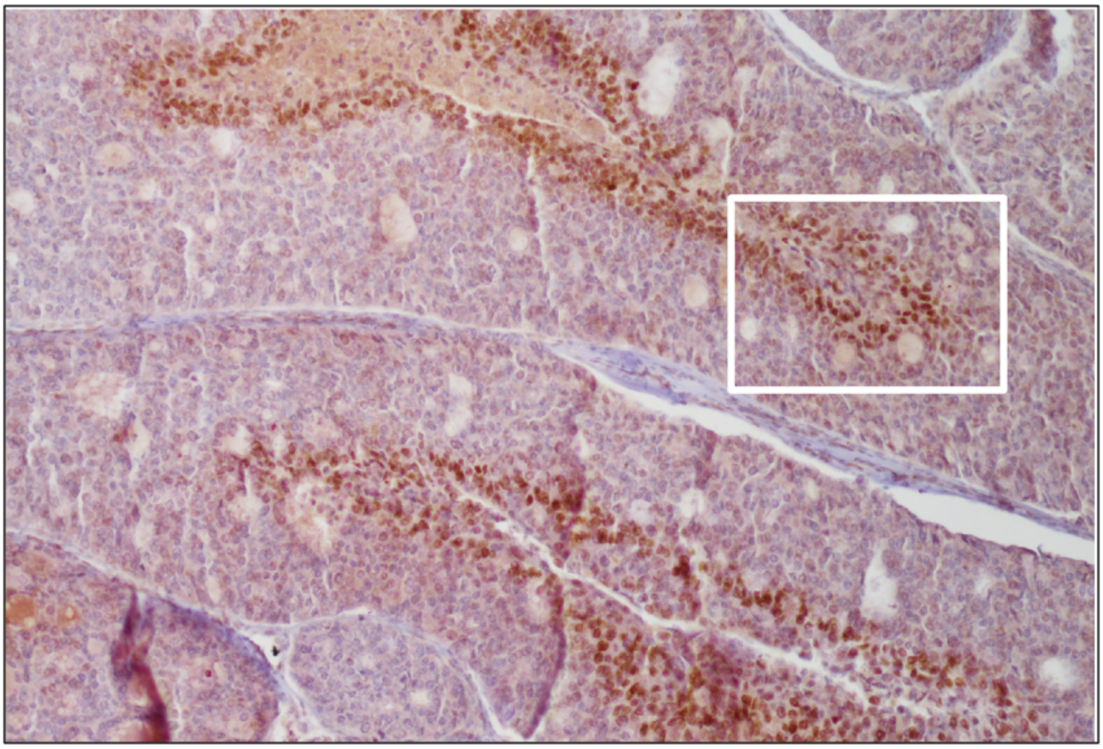

B

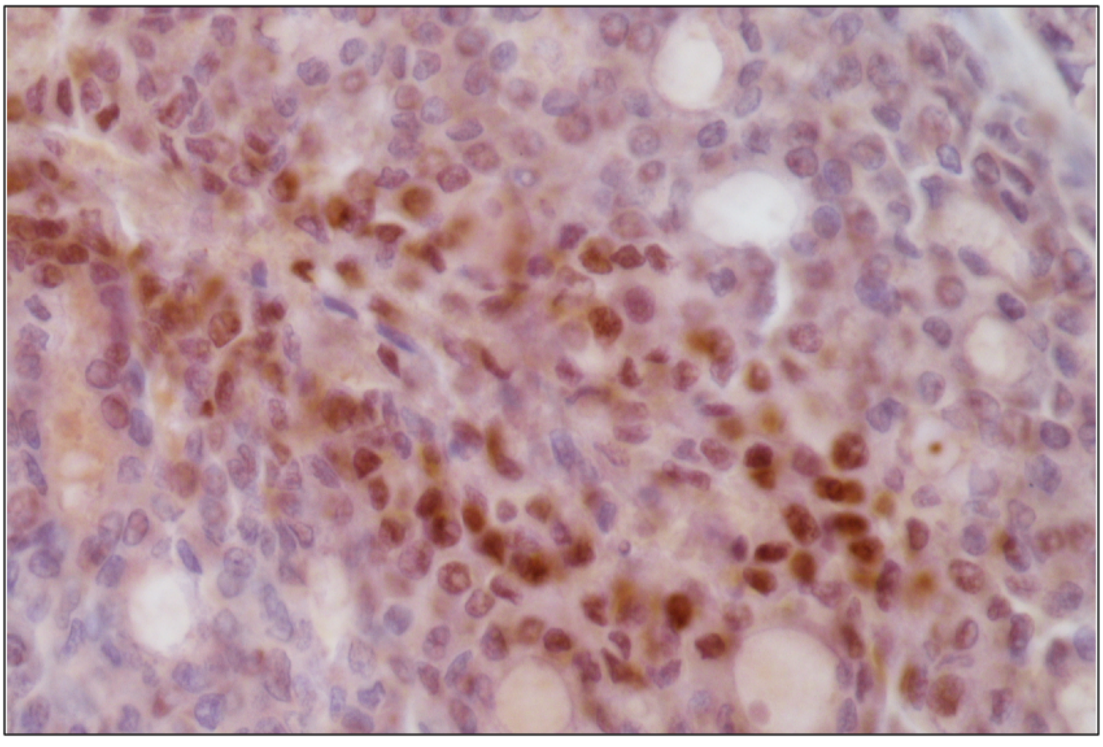

Supplementary_ Figure 4

A

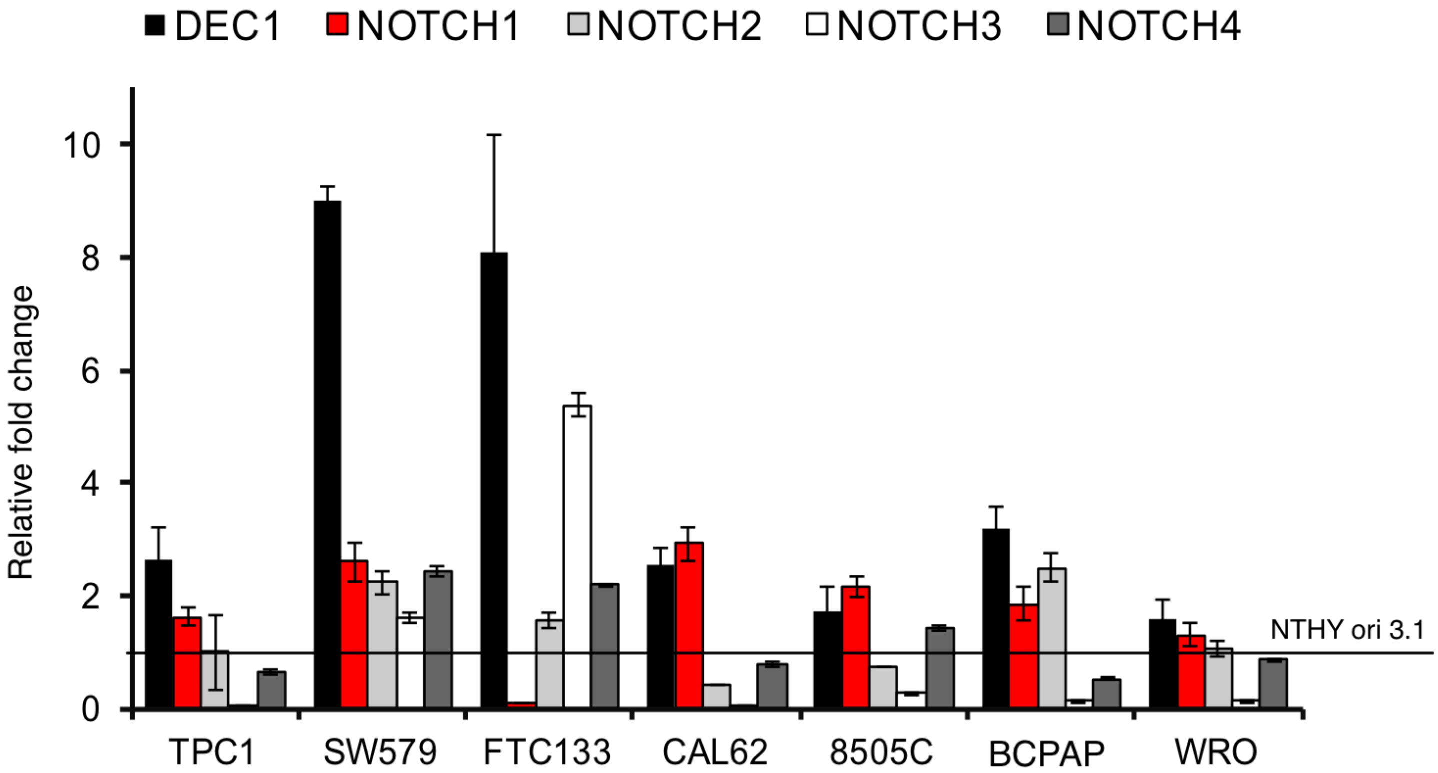

B

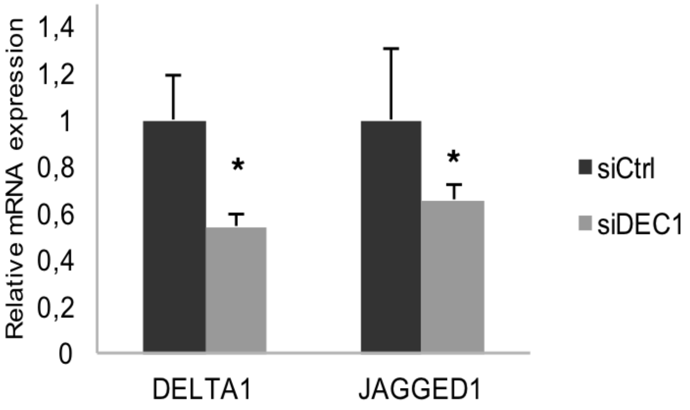

C

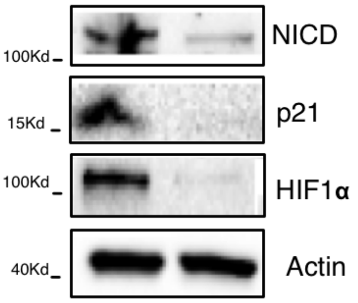

Supplementary Figure 5

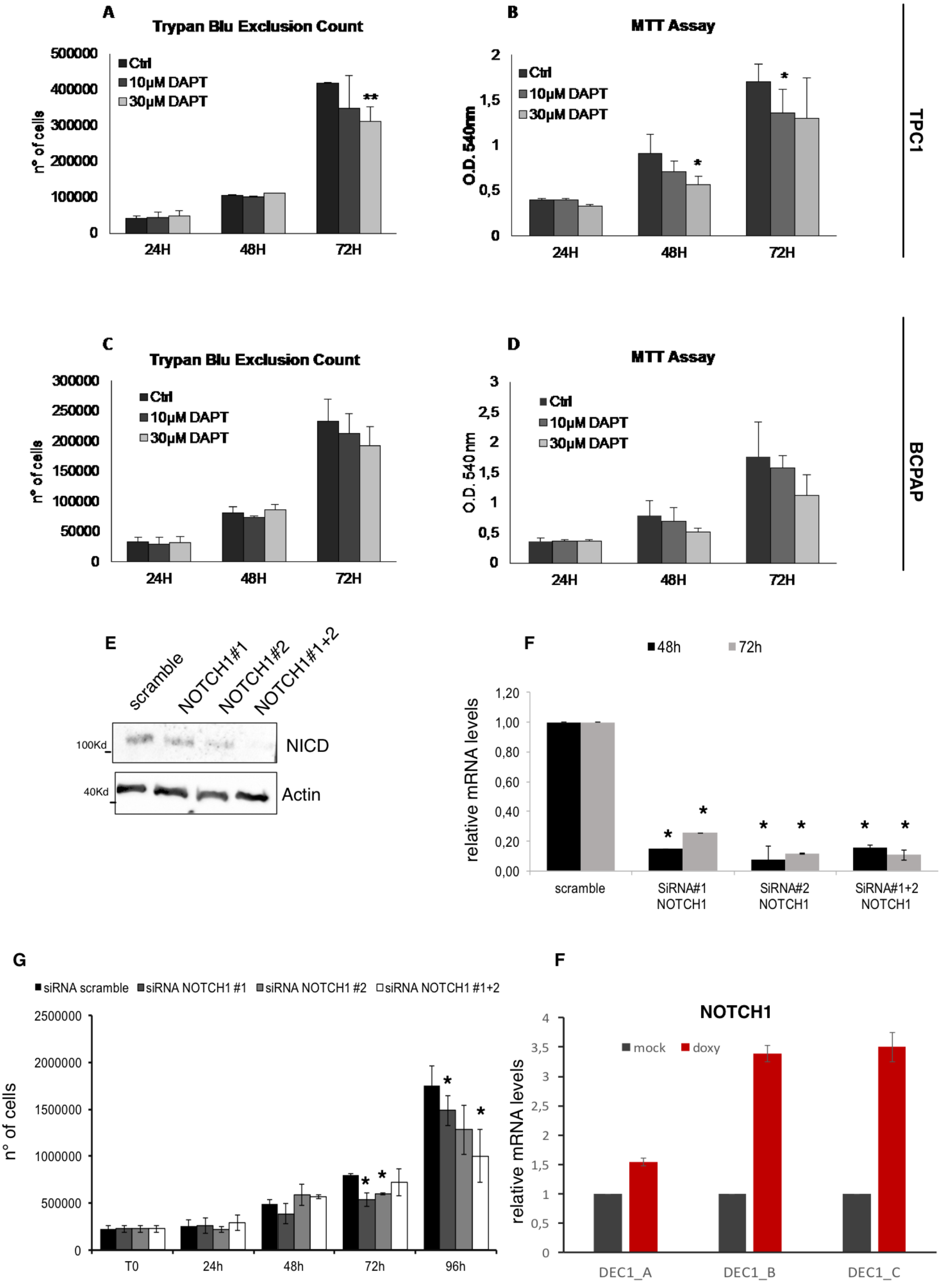

Supplementary Figure 6

A

NOTCH1 IHC	*Positivity Score*	*Number of Positive Tumors*
	≤30	15
	30-50	8
	>50	20
	NA	12

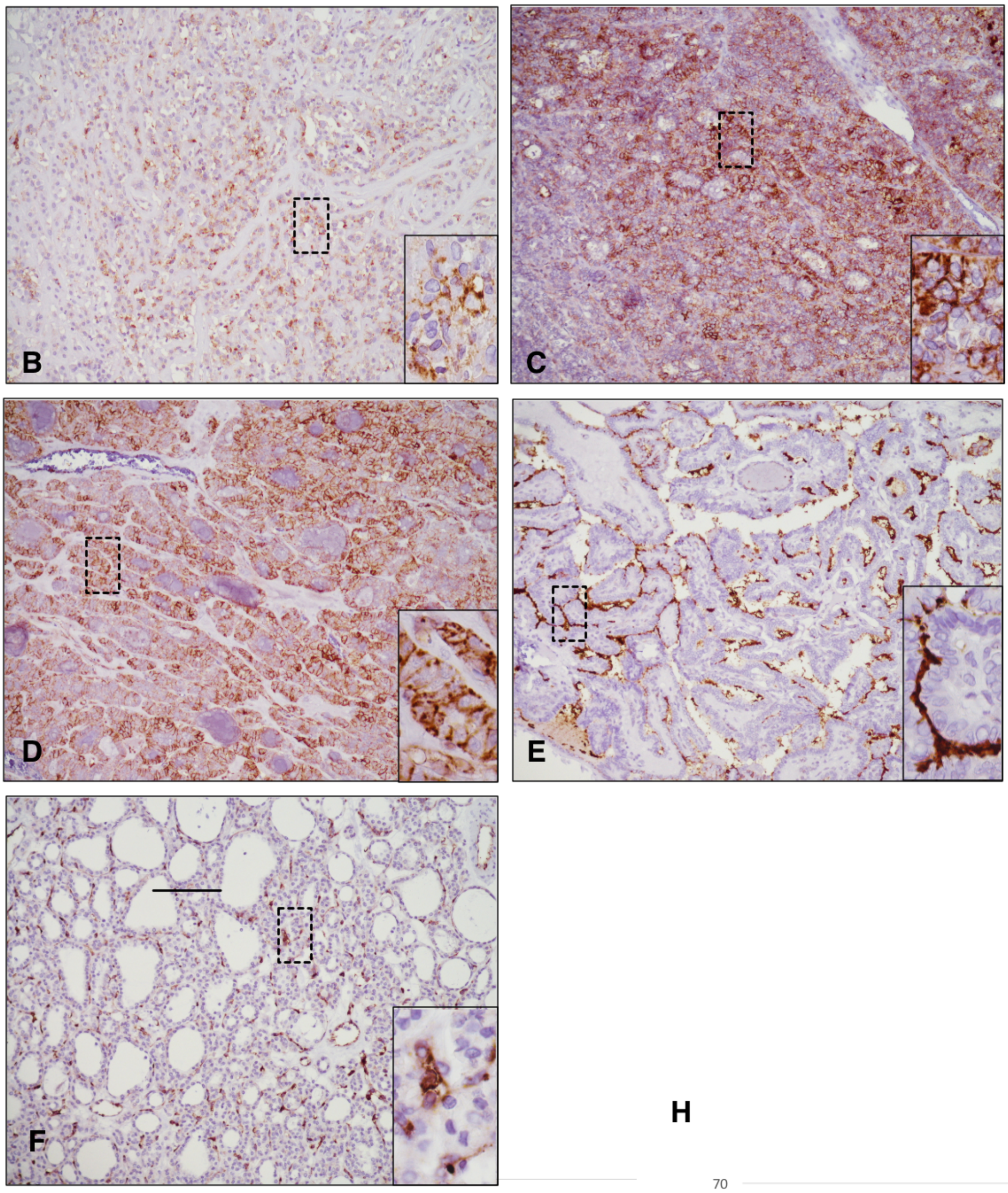

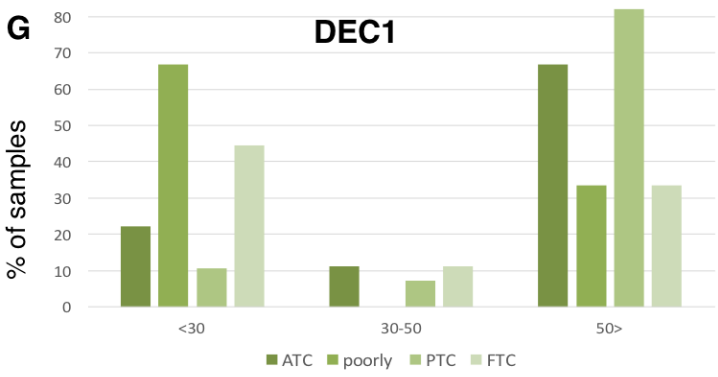

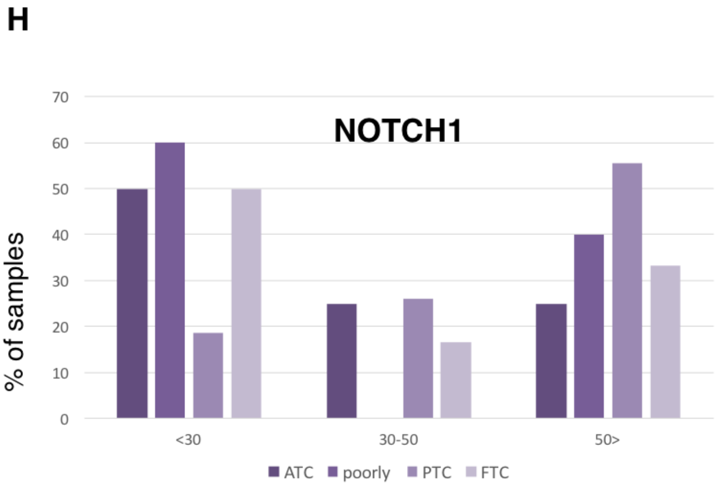

Supplementary Figure 7

A

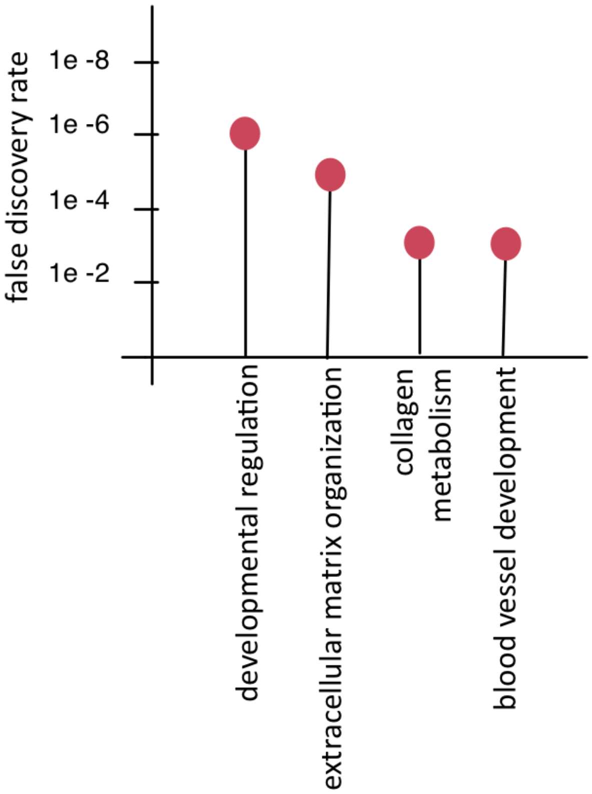

C

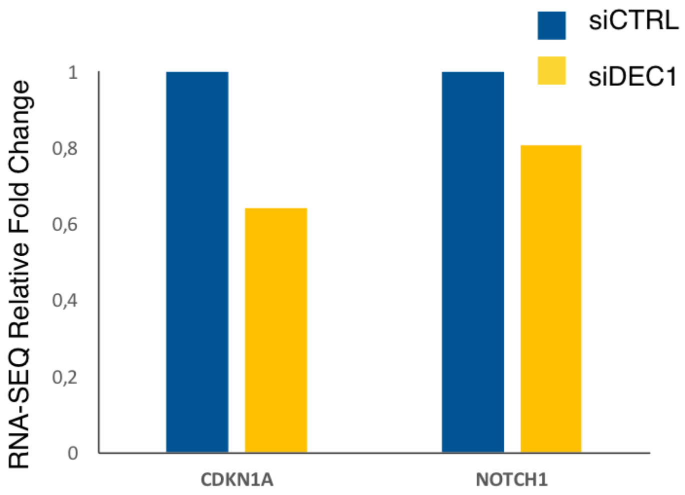

B

Developmental regulation			Extracellular Matrix Organization	Collagen Metabolism	Blood Vessel development
ACVR2B	CRMP1	PAK3	COL12A1	COL12A1	HEG1
ADAM19	CRYAB	PAX2	ITGB8	P3H4	ANTXR1
ADGRG1	ECM1	PTHLH	P3H4	COL5A2	ACVR2B
ANTXR1	EPHB2	PYGO1	COL5A2	FURIN	TGFBR3
APBB2	F2RL1	RAPGEFL1	FURIN	COL5A1	ITGB8
ARF4	FJX1	SLC38A2	COL5A1	COL4A4	EPHB2
ATN1	FURIN	SMPD1	COL9A2	CTSO	CCL2
BHLHE41	GBP3	SPOCK1	COL4A4		UNC5B
BMP1	GLI3	TGFBR3	ADAM19		ECM1
C5AR1	HEG1	TLR4	CTSO		GLI3
CAV1	INHBA	UNC5B	BMP1		COL5A1
CCL2	ITGB8	WNT5A	APBB2		CAV1
CD109	KDM6B	YBX3	COL16A1		ADGRG1
COL12A1	KLF10	ZNF703			
COL4A4	LHX1				
COL5A1	MBOAT7				
COL5A2	MXRA8				
COL9A2	NTN4				

SUPPLEMENTARY FIGURE LEGENDS

Supplementary Figure 1. Downregulation of DEC1 and DEC2 by specific siRNAs. TPC1 and BCPAP were transfected with 30nM of each siRNA and DEC1 downregulation was evaluated over time by qRT-PCR (A-B) or after 72h by Western Blotting (C). DEC2 downregulation was also assesed by qRT-PCR (D-E) and by Western Blotting (F).

Supplementary Figure 2. Effect of DEC1 and DEC2 down-regulation on TPC1 and BCPAP. The viability of cells was evaluated by MTT assay after DEC1 or DEC2 silencing both in TPC1 (A) and in BCPAP (B). The ability to form colonies was evaluated performing the colony forming assay and the size of the colonies was evaluated in TPC1 (C).

Supplementary Figure 3 Representative images of the heterogeneous expression of DEC1 in pre-necrotic area (A-B).

Supplementary Figure 4. A) qRT-PCR analysis of NOTCH1-4 mRNAs, along with DEC1 mRNA in the indicated thyroid cancer cell lines. The histograms represent the fold expression of each transcript in the indicated cell lines relative to their levels in the NTHY 3.1ori (line). B-C) DEC1 knock-down affected NOTCH1 pathway. The expression of NOTCH1 ligand was assessed by qRT-PCR (B) and the expression of NOTCH1 target genes was evaluated by Western Blotting (C).

Supplementary Figure 5. The effect of NOTCH1 pharmacological inhibition by DAPT was evaluated in TPC1 and BCPAP by Trypan blue exclusion count (A-C) and by MTT (B-D). E-F) qRT-PCR (F) and western blot analysis (h48) (E) of NOTCH1 siRNAs silencing efficiency in TPC1. Two different oligos (NOTCH1#1 e NOTCH1#2) as well as their combination were used. G) The effect of NOTCH1 silencing on cell proliferation by Trypan blue exclusion count. H) qRT-PCR analysis of NOTCH1 expression in DEC1 overexpressing clones.

Supplementary Figure 6. A) Summary of IHC analysis of NOTCH1 expression in thyroid cancer samples. B-F) Representative images of NOTCH1 staining in ATC (B), PDTC (C), PTC (D), FTC (E), and FA (F). G-H) distribution of DEC1 (G) and NOTCH1 (H) positivity in the indicated

histotypes of thyroid cancer. The bars represent the percentage of DEC1 positive and NOTCH1 positive samples in each staining category for each histotype. (anti-Notch1 D1E11-Cell Signaling)

Supplementary Figure 7. A) Gene Ontology enrichment analysis of the genes found up-regulated upon DEC1 silencing in thyroid cancer cells. B) Lists of genes for each enriched pathways. C) p21 and NOTCH1 levels in DEC1 silenced and control cells from the RNA-Seq analysis.

SUPPLEMENTARY MATERIALS AND METHODS

Reagents

The Silencer® Select Pre-designed siRNA targeting DEC1 or DEC2 and the Silencer® Select negative control siRNA were purchased from Ambion (Thermo Fisher Scientific). Lipofectamine RNAiMAX, Lipofectamine 2000 were purchased from Invitrogen by Thermo Fisher Scientific. Optimem, Puromycine, DMEM GlutaMAX medium and FBS (fetal bovin serum) were purchased from Gibco by Life technologies. MitomycinC from *Streptomyces caespitosus* was purchased from Sigma Aldrich as powder and solubilized in PBS 1X. Primers were synthesized by Eurofins Genomics. GoTaq polymerase was purchased from Promega. Phusion Polymerase from Invitrogen and Sso Fast EvaGreen Super Mix and iScript cDNA Synthesis Kit from Biorad. Plasmid vector pSG213 was kindly gifted from Prof. R. Tupler (University of Modena and Reggio Emilia), whereas N-terminal p3XFLAG-CMV was obtained from Sigma Aldrich. Kits for DNA extraction and purification were purchased from Machery-Nagel. Doxycyclin was purchased from Sigma Aldrich as ready to use solution, whereas DAPT (N-[N-(3,5-Difluorophenacetyl)-L-alanyl]-S-phenylglycine t-butyl ester) as powder and was solubilized in DMSO.

Clones isolation

For stable clone derivation, 4 x 10^5 BCPAP were transfected with 2.5 µg DEC1 or DEC2-pSG213 or with the empty vector using 7,5µl of Lipofectamine 2000 in a 6well multiplate. 24 hours after

transfection cells were seeded in a p10 plate and Puromycine was added to the medium at the final concentration of 0.5 µg/ml. After selection, single clones were picked, expanded and the overexpression of DEC1 was assessed by Doxycycline induction.

RNA extraction and qRT-PCR analysis

Total mRNA was collected using Maxwell® RSC simplyRNA Cells (Promega) and retro-transcribed using iScript cDNA Synthesis Kit. Quantitative PCR was carried out using the Sso Fast EvaGreen Super Mix in the CFX96 Real Time PCR Detection System (Bio-Rad).

Western Blotting

Cells were lysed in 2X Laemmle Buffer (Bio-rad), the total lysate was frozen and boiled to break membranes. The lysate volume was normalized on the number of cells. Equal amounts of proteins for each sample were resolved on 10% sodium dodecyl sulfate–polyacrylamide gel electrophoresis using the Bio-Rad Mini-Protean apparatus. Gel was blotted onto nitrocellulose membrane using the Trans-Blot Turbo, transfer system, Bio-rad. Following blocking with 5% non-fat milk powder (wt/vol) in Tris-buffered saline (10mM Tris–HCl, pH 7.5, 100mM NaCl, 0.1% Tween-20) for 1h at room temperature, membranes were incubated with primary antibodies directed against the following human antigens: β-actin, anti-FLAG, NOTCH1 (Sigma Aldrich), NOTCH1 (D1E11-Cell Signaling) DEC1 (Bethyl Laboratories) DEC2 (Santa Cruz Biothecnology), total and phospho-AKT (Thr308 and Ser473), p21, HIF1α (all purchased from Cell Signaling Technology, Danvers, MA). The antibodies were diluted in 2% bovine serum albumin–Tris-buffered saline–0.1% Tween according to the manufacturer's instructions. The bound antibodies were visualized by horseradish-peroxidase-conjugated secondary antibodies and an enhanced chemiluminescence detection system, Clarity from Bio-rad.

Colony formation assay

This assay is an *in vitro* cell survival assay based on the ability of a single cell to grow into a colony. Briefly, $1x10^3$ TPC1 or BCPAP knocked-down for DEC1 or DEC2 were seeded on a 100 mm plate in complete medium. After 10 days colonies were fixed in 4% PFA (paraformaldehyde), washed with water and stained with Crystal Violet 0.2% in H_2O. The number and the size of colony were manually counted.

Adhesion Assay

We performed a static adhesion assay able to detect how many cells acquire or lose the ability to adhere to a collagen substrate after particular changes. In our cases we evaluated the ability of TPC1 or BCPAP knocked down for DEC1 or DEC2 to adhere on a pre-coated collagen surface. We pre-coated the surface of a 24 wells multi-plate with 0.1 % of Collagen type IV. Then we discarded collagen exceeding and seeded cells in complete medium. After 30 minutes, we washed cells 3 times with PBS1X to eliminate not adherent cells. Then we fixed cells in 4% PFA (paraformaldehyde) and we stained cells with 0.2% CV. FACS data were analyzed by the FACSDiva6 software. ImageJ software was used for cellular assays quantification.

		DEC1 IHC (total n=52)			
DEC1 positive cells (%)		≤30	30-50	>50	
					pValue
Age					
	<45	4	2	9	0.69
	≥45	10	2	25	
Gender					
	Female	9	4	22	0.64
	Male	5	0	12	
Histological Diagnosis					
	PTC	3	2	23	0.04
	FTC	5	1	3	
	PDTC	4	0	2	
	ATC	2	1	6	
Stage TNM					
	I	7	3	22	0.15
	II	4	0	2	
	III				
	IV	2	1	10	
	NA	1	0	0	
Tumor size (cm)					
	≤5	9	3	30	0.17
	>5	4	1	4	
	NA	1	0	0	
Metastasis					
	yes	2	1	14	0.22
	no	12	3	20	
Metastasis site					
	Single	1	1	9	0.39
	Multiple	1	0	5	
	NA	12	3	20	

Supplementary Table I correlation of DEC1 expression with clinical pathological features of thyroid cancer

Supplementary Table II List of primers

DEC1_F	CCTTGAAGCATGTGA
DEC1_R	CATGTCTGGAAACCT
DEC2_F	TAACCGAGCAACAGC
DEC2_R	GCATGTTTGAAATCC
NOTCH1_F	AAGTGTGAAGCGGCCAAT
NOTCH1_R	CATGTCCCGGCGTTCTTG
NOTCH2_F	CAGGCACGTCAGGGGTTAAT
NOTCH2_R	TCCTGGTGAGCAGACACAAC
NOTCH3_F	GCCAAGCGGCTAAAGGTAGA
NOTCH3_R	TGAGTCCACTGACGGCAATC
NOTCH4_F	CCCCCAAAATGAAGGGTTGTG
NOTCH4_R	CACGTGGAAGATGTCTGCTCT
p21_F	CATGGGTTCTGACGGACATC
p21_R	TGCCGAAGTCAGTTCCTTGT
MYC_F	ACTCTGAGGAGGAACAAGAA
MYC_R	TGGAGACGTGGCACCTCTT
DELTA1_F	CAGGCCATCTGCTTCACCA
DELTA1_R	AAGACGATACCCACAGTGCCC
JAGGED1_F	CGGGAACATACTGCCATGAAAATA
JAGGED1_R	ATGCACTTGTAGGAGTTGACACCA
HEY1_F	CTGCAGATGACCGTG
HEY1_R	GGCATTCCCGAAATC
GUSB_F	TTGAGCAAGACTGATACCACCTG
GUSB_R	TCTGGTCTGCCGTGAACAGT
CWC27_F	TCCAAGGGAAATTAAAAGGCTGA
CWC27_R	TCTTCCTCAGCTTCCTCTCCA
VRK1_f	TGGCAAATTGGACCTCAGTGT
VRF1_R	ACACCAGGTTCCTTGCTTTCT
BUB1_F	GCCATCAAGCCCAAGACTGA
BUB1_R	ATCTCCCTGGGTAGCTTCGT
LMNB1_F	CAAGTGCAAGGCGGAACAC
LMNB1_R	TCTCGAAGCTTGATCTGGGC
AURKB_F	CTGCTCTTAGGGCTCAAGGG
AURKB_R	GTAGTCCAGGGTGCCACAC
CENPM_F	TTGCCCTCCAGTGTGAATCG
CENPM_R	AGAAGCTGGCATCCACATGG
NUF2_F	TCAGAGAAGCATGCCGTGAA
NUF2_R	TGTGCGGCGTTTAACTGTTG

ARFIP1_F	AGAGGGTGTTATTGAAGCAGGA
ARFIP1_R	ATCACTTCCTTGCTGAGCCA
PARP1_F	CGGAAGCTGGAGGAGTGAC
PARP1_R	CCCCTTGCACGTACTTCTGT
PSMA4_F	GGAGCCAATACCTTGTGAGCA
PSMA4_R	TCCCAGCCAATGTACAGCAA
LGR5_F	AGTCAGCTGCTCCCGAATC
LGR5_R	CAGTGAATGCTCCCTTGGGA
HSP90AA1_F	CCCAGACACATGCTAACAGGA
HSP90AA1_R	AGGGGTGGCATTTCTTCAGT
PTTG1_F	CTGTAAAGACCAAGGGACCCC
PTTG1_R	TGAGGCAGGAACAGAGCTTT
ASF1B_F	CGGTTCGAGATCAGCTTCGA
ASF1B_R	ACCAGCACCGAGTCTAGGAT
UBE2C_F	CCTTGAACACACATGCTGCC
UBE2C_R	CTGGTGACCTGCTTTGAGTAG
UBE2T_F	TGCTGGAAGGATTTGTCTGGA
UBE2T_R	AGAGGTCAACACAGTTGCGA
